# Q&A: Single-molecule localization microscopy for biological imaging

**DOI:** 10.1186/1741-7007-8-106

**Published:** 2010-08-11

**Authors:** Ann L McEvoy, Derek Greenfield, Mark Bates, Jan Liphardt

**Affiliations:** 1 Biophysics Graduate Group, University of California Berkeley, Berkeley, CA 94720, USA; 2 Physical Biosciences Division, Lawrence Berkeley National Laboratory, Berkeley, CA 94720, USA; 3 Department of NanoBiophotonics, Max Planck Institute for Biophysical Chemistry, Göttingen 37077, Germany; 4 Department of Physics, University of California Berkeley, Berkeley, CA 94720, USA; 5 LS9, Inc., 600 Gateway Blvd, South San Francisco, CA 94080, USA

## Why is it important to understand how cells are organized? 

Prokaryotic and eukaryotic cells possess a complex internal structure, including protein networks, genetic material, internal and external membranes and organelles. These elements provide physical structure to cells, and a means to localize particular biochemical processes to specific cellular regions. The structure of the cell is intimately linked to its biological functions, and hence the study of the physical structure and organization of the cell is a valuable means of gaining insight into cell biology.

## How do biologists typically visualize the spatial organization of cells? 

Light microscopy and electron microscopy (EM) are widely used in cell biology to observe the small details of biological samples. In the past decade, the development of new fluorescence microscopy methods has revolutionized how biologists use light microscopes to study cellular structure. However, a significant disadvantage of fluorescence microscopy is its spatial resolution, or image sharpness. Although the structures of the protein complexes within the cell exist at length scales of micrometers to nanometers, the light microscope is unable to resolve structures smaller than approximately 250 nanometers. Features smaller than this size appear blurred in the microscope image. This 'resolution limit' arises as a result of the diffraction of light and leaves many cellular structures difficult or impossible to observe.

EM allows for much higher-resolution images than light microscopy. However, unlike light microscopy, which has the advantage of excellent fluorescence labeling specificity, EM lacks powerful and easy labeling strategies. In addition, EM imaging can only be performed on fixed samples and often requires harsh sample preparation techniques that can disrupt native protein structures. Ideally, we would use techniques that combine the specificity of labeled probes with the resolution of EM.

## What is single-molecule localization microscopy? 

Taking advantage of sensitive fluorescence detection methods, single-molecule imaging techniques have improved our understanding of the structure and function of proteins. Recently, these methods have been applied to high-resolution light microscopy, allowing light microscopes to take images with a spatial resolution far beyond the diffraction limit. It was discovered that by imaging individual fluorescent molecules one at a time, an image of a fluorescently labeled sample can be reconstructed at much higher resolution than previously possible. For the purposes of this review, we will refer to this method as single-molecule localization microscopy (SMLM), as it is based principally upon single molecule detection and localization. SMLM combines the benefits of both fluorescent light microscopy and EM, producing nanometer-resolution images of structures that have been labeled with high specificity.

Various implementations of SMLM have been developed by different research groups, and as a result the technique is known by several other names, which include photoactivated localization microscopy (PALM), stochastic optical reconstruction microscopy (STORM), and fluorescence photoactivation localization microscopy (fPALM) [1-5].

## How does SMLM work? 

**Figure 1:**
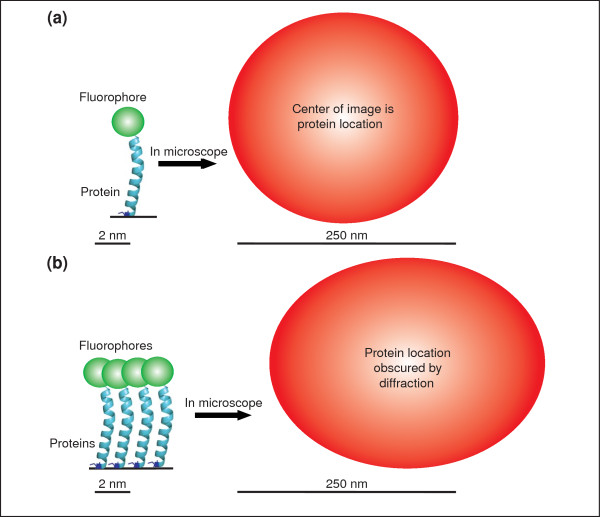
**The images of fluorophores observed with a microscope are blurred by the wave-like properties of light**. **(a) **The image of a single fluorophore (red circle) has a width greater than approximately 250 nm when viewed with visible light, despite the fact that the fluorophore itself is only a few nanometers in size. The image of such a point emitter is called the point-spread function (PSF). The position of the fluorophore in this case can be determined by measuring the center position of the image, which is equivalent to the PSF in this case. **(b) **When multiple fluorophores are located in close proximity, their images overlap and it becomes difficult to distinguish the individual fluorophores from one another. It is the width of the PSF that limits the ability of the microscope to resolve closely spaced fluorophores. The fluorophore positions cannot be determined accurately in this case.

A single fluorophore inside a cell behaves as a single point source of light. However, when viewed through a microscope, the size of the image of the fluorophore is much larger than the size of the fluorophore itself (Figure 1). The broadening of the image of a point source is due to diffraction, an optical effect resulting from the wave-like properties of light interacting with the optics of a microscope; this effect limits the spatial resolution of conventional optical microscopy to around 250 nm laterally, and around 500 nm along the optical axis. The broadened image of a point source produced is termed the point-spread function (PSF) of the microscope (Figure 1a, right).

Although the image of the fluorophore is broadened by diffraction, the center of the observed image corresponds to the position of the fluorophore. When only a single fluorophore is emitting light, the position of the fluorophore can be found very precisely by measuring the center position of its image. Therefore, if only one tagged protein were present inside the sample, we would be able to know the position of the protein to high precision (Figure 1a).

**Figure 2:**
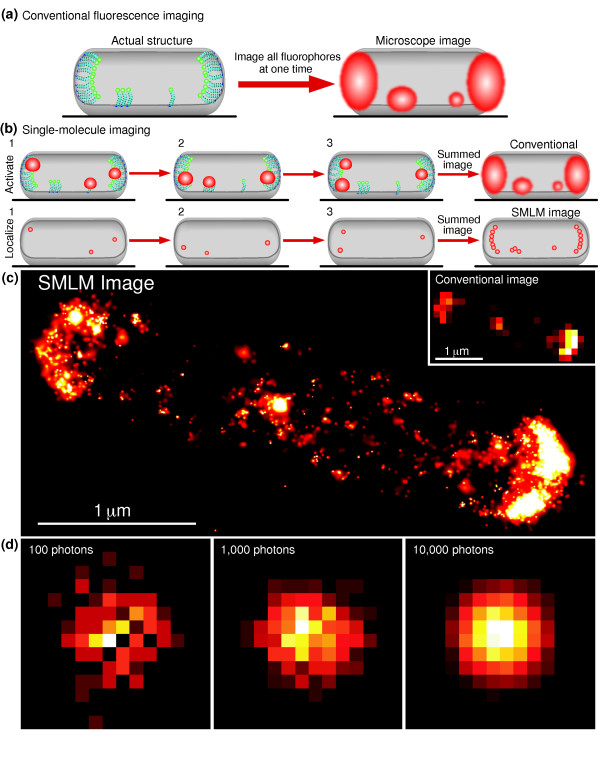
**Principles of single molecule localization microscopy**. **(a) **Conventional fluorescence microscopy excites all fluorophores at once, and therefore the images of closely spaced fluorophores overlap. In this case, the best possible image resolution is around 250 nm when using visible light. **(b) **Single molecule localization microscopy (SMLM) techniques activate and observe only a sparse subset of fluorophores at any given time. Because the images of each fluorophore no longer overlap, the location of each fluorophore can be determined precisely. The fluorophore positions can be used to create a super-resolution image of the sample. Instead of plotting the diffraction-limited image of the fluorophore (top sequence), the measured location of each fluorophore is plotted (bottom sequence). **(c) **SMLM image of tagged chemotaxis receptors in *Escherichia coli*. Each small point is a single fluorophore with approximately 15-nm localization precision. The SMLM image is much sharper than the conventional image (inset in (c)). **(d) **The location of fluorophores can be determined more precisely if the fluorophore emits more photons. If the fluorophore only emits 100 photons (left) it becomes more difficult to locate the center of its image in comparison to emission of 1,000 (middle) or 10,000 (right) photons.

In cells, many proteins exist in dense complexes, such that the distance between each protein is less than the wavelength of the light used to image them. This means that closely spaced labeled proteins (closer than 250 nm) appear as a single fluorescent entity when viewed through the microscope (Figure 1b). In this situation, it becomes difficult to distinguish the individual fluorophores, and it is impossible to observe the spatial organization of the sample for length scales smaller than several hundred nanometers. This is the reason that traditional fluorescence microscopy, which illuminates all fluorophores in the sample simultaneously (Figure 2a), is limited in its spatial resolution.

Since it is difficult to spatially resolve closely spaced fluorophores, SMLM uses the innovative approach of separating the fluorescence of each emitter in time. Instead of imaging all the fluorophores simultaneously, SMLM techniques image each individual fluorophore one at a time, making it possible to find the position of each molecule with high precision. Once all of the positions have been found, they are plotted as points in space to construct an image. The spatial resolution of this image is not limited by diffraction, but only by the precision of the localization process for each fluorophore.

To observe each protein individually, photoactivatable fluorophores are used. These are fluorescent molecules for which the fluorescence emission can be switched on and off under the control of an external light source. The activation light source illuminates the entire sample but at such a low intensity that only one or a few fluorophores are activated at a time, and the fluorophores that are activated at a given time is random. This enables different photoactivatable fluorophores to be 'turned on' at different times, and allows the image of each fluorescent label to be observed individually. Computer algorithms are used to find the locations of each molecule, and these fluorophore locations are then assembled into an image (Figure 2c). The location of the molecule is determined by finding the centroid of the image obtained from each molecule (discussed in detail later). The precision of the position measurement is dependent on how bright the fluorophore is over the background signal. The brighter the fluorophore, the easier it is to determine its location (Figure 2d).

SMLM imaging time is limited by how quickly it is possible to turn on each fluorophore and then turn it off. To image quickly, it is often necessary to use high excitation power so that each fluorophore is turned off immediately after excitation. Because SMLM techniques image each fluorophore individually, as the sample density increases so does the time required to take an image.

## Why would I use SMLM? 

SMLM has many benefits over traditional imaging techniques. This method allows proteins of interest to be labeled specifically and provides approximately ten times higher spatial resolution than traditional fluorescence light microscopy. It is therefore useful for observing biological structures at the nanometer scale, and for examining the molecular structure of protein complexes [1-5].

Many biologists are interested in understanding how proteins interact inside cells. However, because of the resolution limitations of standard fluorescence microscopy, it is only possible to identify protein co-localization to within around 250 nm. Because single-molecule techniques obtain images of higher resolution, it is possible to co-localize two proteins to around 25 nm, allowing for much more accurate co-localization experiments [6,7].

In addition, SMLM can be used to track how single proteins move inside cells. Individual protein positions can be assembled into tracks that show how populations of proteins move in cells over time, on the nanometer scale [8].

## I would like to take an SMLM image of proteins within a cell. Should I? 

Single-molecule imaging is more complicated than conventional fluorescence imaging. It is computationally intensive and requires the use of different fluorophores, many of which are not well characterized. Ideally, the researcher would start with a system that has been successfully labeled and imaged previously using either fluorescent proteins or immunofluorescence methods. Starting with such a system will confirm that the system can be labeled and will give insight into the best labeling strategy (that is, is a linker necessary in the case of a fluorescent protein label; should the amino or the carboxyl terminus be tagged; should fluorescent antibodies be used?). Furthermore, imaging problems are easier to troubleshoot when the typical cellular localization of the protein of interest is already known.

**Figure 3:**
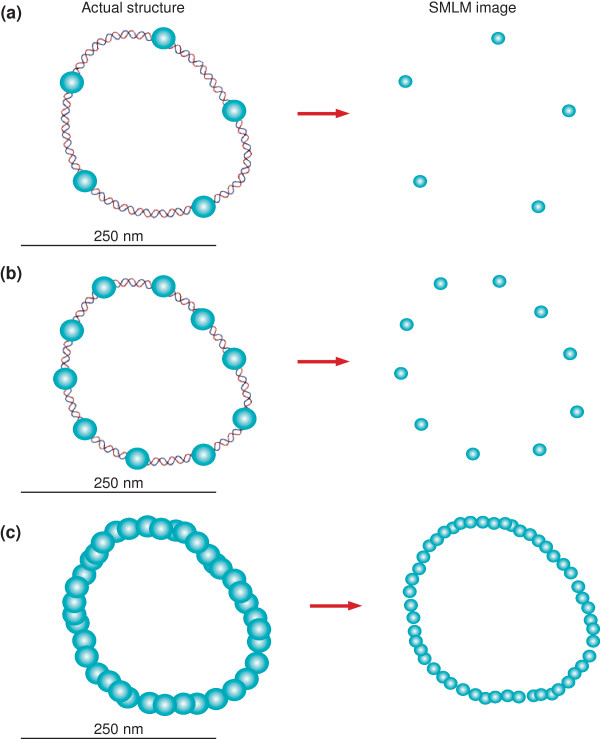
**Higher labeling densities increase the amount of detail observed in SMLM imaging**. In this example, the structure of a small loop of DNA is determined by labeling the DNA with fluorophores (left column) and determining the fluorophore positions with SMLM (right column). The detail in the resulting image of the DNA (right column) is only as good as the labeling density. **(a) **Labeling the DNA with only five fluorophores (left), does not preserve the actual structure of the DNA (right). **(b) **By doubling the number of fluorophores labeling the DNA (left), the structure of the DNA loop starts to appear (right). **(c) **By densely labeling the structure (left) the shape of the DNA becomes apparent (right).

On the basis of previous studies, it may be known how fixation affects the sample structure. If not, it is important to test different fixatives to ensure that the protein complex of interest can be chemically fixed without perturbation. Some fixatives preserve some protein complexes better than others, so it is necessary to check which ones are best for a particular system. It is also very important to have an assay for functionality to verify that the attachment of a fluorescent tag does not perturb the protein of interest. In addition, as in all single-molecule experiments, it is necessary to decrease background fluorescence signals by using non-fluorescent imaging media and by using clean coverslips to increase the signal-to-noise ratio obtained from a single emitter. It is also important to have densely labeled samples. The sharpness of the reconstructed image is directly related to the labeling density. It becomes increasing difficult to observe fine detail if the labeling density is low (Figure 3).

## Practically speaking, how do I prepare a sample for single-molecule imaging? 

Single-molecule imaging requires the use of photoactivatable or photoswitchable fluorophores, of which there are two main categories: photoactivatable fluorescent proteins (paFPs), and photoswitchable synthetic fluorescent dye molecules such as Cy5 [4,9,10]. As with traditional fluorescent proteins such as green fluorescent protein (GFP), paFPs can be genetically encoded and fused to proteins of interest. Photoswitchable dyes can be conjugated directly to proteins of interest, or can be conjugated to antibodies that target the protein of interest (immunofluorescent labeling). The choice of dyes or paFPs depends on the biological application. paFPs have the advantage of labeling each protein of interest directly, so they are highly specific. However, paFPs are dimmer than dyes and multicolor imaging is more challenging because many paFPs have similar emission spectra. Some commonly used paFPs include mEos2, pamCherry, Dronpa and Dendra2. Synthetic dyes, by contrast, are very bright but it can be difficult to label proteins with dyes, particularly in living samples. Immunofluorescence techniques are dependent on the quality of the antibodies used and often have higher background signal as a result of nonspecific staining. They also often have a lower density of labeling in comparison to paFPs. Samples labeled with paFPs can be imaged in any non-fluorescent media, whereas some synthetic dyes require the use of reducing agents in the imaging buffer to photoswitch properly [4,9,10].

To acquire an image of a sample labeled with paFPs, it is necessary to first grow the cells and express the fusion protein. Once the cells have been grown, they should be fixed and either placed on a coverslip for imaging, or imaged on the coverslip they were grown on. Alternatively, if dyes are used, the cells should first be grown and then fixed. The cells are then permeabilized and labeled using a strategy such as immunofluorescent labeling.

Because SMLM image acquisition may take a long time, any drift of the microscope stage during data collection will need to be corrected. For this purpose, it is often useful to include fluorescent particles on the surface of the sample or the glass substrate. These fluorescent particles, such as gold nanoparticles, allow you to track any lateral movements of the stage during image acquisition and correct for drift in software.

## What equipment do I need to build such a microscope? 

In general, conventional fluorescence microscopes can easily be modified for SMLM. In most cases, SMLM has been carried out using total internal reflection (TIR) illumination, which limits the light to the bottom 100 to 150 nm of the sample, thus reducing out-of-focus light and making it easier to observe single molecules. It is convenient to use TIR imaging if you are imaging proteins close to the bottom of cells. However, for thin samples such as EM sections or small cells, it is possible to illuminate using epi-fluorescence.

To photoactivate and excite fluorophores in the sample, it is necessary to add the appropriate laser lines to an existing microscope. The choice of the lasers used depends on the activation and excitation spectra of the fluorophores. Lasers are frequently utilized because they deliver the necessary power to image quickly. Like all fluorescence microscopy, it is necessary to have the appropriate excitation and emission filters to maximize your signal-to-noise ratio [10,11]. It is beneficial to use an objective with a high numerical aperture (NA = 1.4 or higher) so that as many photons as possible are collected. To collect the data, a sensitive CCD camera (such as an electron-multiplying CCD) is also required to observe as many photons as possible. Because single-molecule imaging techniques are wide-field and it may take a long time to look at each fluorophore individually, the data files obtained can become quite large [10,11]; therefore, a fast enough computer with sufficient storage space is essential.

## How do I convert the raw data to a super-resolution image? 

Once you have acquired your single-molecule imaging data, you will typically have a stack of thousands to hundreds of thousands of single image frames. Each frame will have points of intensity corresponding to the light emitted from a fluorescent label. It is necessary to find the locations of each fluorophore in each frame and then computationally assemble those locations into a composite image. This composite image can be thought of as a map of the best estimation of where the fluorophores are located during imaging. We will consider the case of two-dimensional (2D) imaging for ease of discussion.

To find the location of each fluorophore, it is necessary to first identify each single molecule. This is done by choosing an appropriate threshold to distinguish the signal each molecule emits from the background [10,11]. If the signal is high enough, it is considered to be a target fluorophore. If the switching event lasts longer than one image frame, signals can be combined across frames to increase the signal obtained from each fluorophore. Once a target fluorophore is found, the signal is fitted to a 2 D Gaussian distribution (or the centroid of the signal is determined). How well a Gaussian can fit the signal is dependent on how bright the signal is above background (Figure 2d). In the SMLM image, the location of each fluorophore is represented as a small Gaussian intensity peak, whose width is scaled according to the precision of 'localizing' that fluorophore. In other words, the blurred image of the emitter is replaced with the best guess as to where the fluorophore is located. As it may be necessary to image the sample for a long time, it is also important to perform drift correction on the image using appropriate methods [1,5,10].

Image processing is a challenging aspect of single-molecule imaging. Recently, a new ImageJ plug-in was developed to process single-molecule imaging data in both two and three dimensions [12]. The development of such processing tools will facilitate the use of single-molecule imaging techniques for the broader scientific community.

## Can you generate three-dimensional images? 

Yes, three-dimensional (3D) single-molecule imaging has been carried out using both dyes and paFPs [13,14]. 3 D imaging can be performed using several methods. One approach is to break the axial symmetry of the PSF by adding a cylindrical lens to the imaging path, therefore causing the shape of each fluorophore's image to change depending on its height within the sample. The user can calibrate how the image changes as the sample is moved axially, and use this information to determine the height of the fluorophores in the sample. This technique has a wide z-range (at least 3 μm [15]), but altering the shape of the PSF complicates the localization algorithms and may decrease the lateral resolution of the image [13,15]. A more precise way of getting 3 D information is to use interferometry, which uses phase information from the light emitted by the fluorophore to obtain height information. This allows for 10 nm axial resolution, but because of the limitations of the current system, imaging is restricted to a relatively thin region at a depth of around 500 nm into the sample [14]. Interferometry requires the use of multiple objective lenses, significantly increasing the complexity of the system and making alignment and data processing more challenging.

## Do I have anything more than a pretty picture? 

Because single-molecule imaging techniques look at each molecule individually, in principle it is possible to count each photoactivation event as representing one fluorophore. If the fluorophore is an irreversibly photoactivatable protein (that is, once the protein is observed, it is not capable of re-excitation), the number of excitation events corresponds to the number of proteins observed in the sample. In addition to the number of proteins, you also acquire the location of each protein in the sample. Essentially, a 'protein map' is obtained that can be used to determine the nearest-neighbor distances for all the proteins. It is also possible to search for ordered protein structures; however, the error associated with each protein position may obscure any regular ordered structure depending on the dimensions of the structure [16].

It is important to keep in mind that there are many caveats associated with counting proteins as well as carrying out statistical analysis with single-molecule imaging data. It is important to ensure that only one fluorophore at a time in each diffraction-limited region (around 250 nm) is excited, which requires very low activation power. This extends the time required to image the sample. Also, if you want to count absolute numbers of proteins, it is necessary to image the sample until all the proteins have been activated, excited and then photobleached. Another concern is that there may be a population of paFPs that do not fold properly and are therefore not observable, or that are observable but emit too few photons to be identified as single molecules. Therefore, caution must be taken when making statements about the absolute numbers of proteins in a biological sample, and it is often more practical to draw conclusions about the relative number of proteins within a sample.

## What kinds of biological samples have been imaged with single-molecule imaging techniques? 

So far, the biological samples that have been imaged with SMLM include focal adhesions, microtubules, proteins in cryosections and chemotaxis receptors inside bacteria. All these samples are ideal for single-molecule imaging because they are thin samples or are associated with a flat membrane. They also have little 3 D structure, and can be densely labeled. One 3 D structure that has been imaged using single-molecule imaging techniques is the mitochondrion [15]. Using antibody labeling, it was possible to image the mitochondria with a z-range of 3 μm, and a z-resolution of approximately 50 nm.

SMLM techniques are still quite new, and so only a few studies have used them to understand and model biological processes. Greenfield *et al. *[16] used SMLM imaging to develop a model of how chemotaxis receptors in *Escherichia coli *organize in growing cells. In addition, they confirmed a theoretical prediction that many small clusters of receptors exist inside cells; these small clusters were previously obscured by autofluorescence [16]. Using live and fixed-cell SMLM, Hess *et al. *[17] obtained high-resolution images and dynamic information from influenza hemagglutinin, a clustered membrane protein, to differentiate between membrane organization models in fibroblasts. Another recent study used SMLM to show that there is a protein conformational change in the T-cell antigen receptor on activation [18].

## What if I want to look at living cells? 

It is possible to perform single-molecule imaging on live cells. Live-cell imaging often utilizes paFPs, as the preparation necessary for dye conjugation is more difficult to perform on living samples. Like fixed-cell imaging, live-cell imaging still excites each fluorophore individually; therefore, at any given time interval, only a few fluorophores will be observed [19].

One caveat of live-cell SMLM is that it is relatively slow compared to other fluorescence-imaging techniques. Because each fluorophore is localized at a different point in time, to create a time-lapse movie, the localizations must be binned into time windows and a series of SMLM images are reconstructed. With current techniques, these time windows are typically seconds in duration to obtain a sufficient number of localizations in each window. In addition, care must be taken to avoid cellular damage by reducing laser power, which slows down image acquisition. Therefore, in many cases, the speed of most dynamic biological processes is too fast to be captured by live-cell SMLM movies. Instead, it may be more useful to use SMLM to track the individual movements of proteins inside live cells to nanometer precision [8].

## Can you image deep into tissues? 

It is difficult to image deep into cells with single-molecule imaging techniques. As one images deeper, the cellular autofluorescence increases, which can obscure the signal observed from single molecules. It also becomes more difficult to accurately determine the location of the fluorophores because the image of the fluorophore can change as a result of aberrations in the imaging system and heterogeneity in the sample.

To obtain SMLM images from deep inside cells, it is possible to section tissues to observe thinner samples. Alternatively, temporal focusing can be used in combination with SMLM to image deeper into cells and tissues [20]. Temporal focusing restricts the light used to excite the proteins to a thin sheet, thus eliminating some of the background autofluorescence.

## Really, how difficult is it to do single-molecule imaging? 

Although single-molecule imaging techniques offer better resolution than conventional fluorescence microscopy, they can be complex and time-consuming. Most biological structures are 3 D, and so to make meaningful statements about the structure of protein complexes, 3 D imaging is required. In addition, many interesting protein complexes reside deep within cells. 3 D imaging deep into cells is very difficult using current SMLM techniques, as described above. Another important point is that fine structural details can only be mapped using high-density labeling (Figure 3). In some cases it can be useful to localize sparse individual fluorophores, but to observe nanoscale structures it is necessary to label the sample with a sufficient density of fluorophores, as defined by the Nyquist criterion [19].

Despite these challenges, however, SMLM offers the highest resolution of all current fluorescence microscopy techniques. It is also relatively easy to implement in comparison to other super-resolution techniques.

## What other techniques can acquire images with sub-diffraction-limited resolution? 

Other methods of optically imaging at length scales below the diffraction limit include stimulated emission depletion microscopy (STED) [21] and structured illumination microscopy (SIM) [22]. Both STED and SIM use specific illumination light patterns to achieve a smaller PSF and improved spatial resolution. They are more challenging to implement than SMLM techniques, but are both currently commercially available from the main microscope manufacturers. STED has theoretically limitless resolution, can be done in 3 D, deep into cells, and can be used to image live cells [23]. STED imaging is much faster than single-molecule imaging techniques; however, the speed of the imaging depends on the signal-to-noise ratio within the sample, the sample thickness, and the image size. The brighter the sample, the easier it will be to image quickly and obtain axial information. The first demonstration of video-rate live-cell imaging at sub-diffraction-limit resolution was accomplished using STED, achieving frame rates of 30 Hz at a spatial resolution of 60 nm [23]. Some fluorophores are particularly well suited for STED imaging, including enhanced yellow fluorescent protein (EYFP) and mCitrine, in addition to the dyes Atto 647N and Atto 655.

SIM uses periodically modulated illumination light patterns to generate sub-diffraction-limit images, and can be used for 3 D imaging of thick biological samples using conventional fluorophores. It is much faster than single-molecule imaging techniques, making live-cell imaging highly practical [24]. Complete 3 D reconstructions of thin samples (around 2 μm) can be obtained in 15 to 30 seconds. However, once again, image acquisition times depend on sample brightness and thickness. SIM's main disadvantage is the resolution, which is only twice the resolution of confocal microscopy. In addition, SIM relies on mathematical calculations to convert the raw data into final images; if the sample conditions are not ideal, this can lead to artifacts in the image reconstruction.

Ideally, we would combine several different imaging modalities to understand biological systems. However, like all techniques or assays, it is important to consider which methods are appropriate for a particular system. With the invention of new imaging modalities like SMLM, it will be very exciting to see how they are adopted and applied to biological systems in the future. It may now be possible to examine biological processes, once obscured by the diffraction limit, at a new level of detail.
